# *Crassisporus* gen. nov. (Polyporaceae, Basidiomycota) evidenced by morphological characters and phylogenetic analyses with descriptions of four new species

**DOI:** 10.3897/mycokeys.57.38035

**Published:** 2019-08-21

**Authors:** Xing Ji, Dong-Mei Wu, Shun Liu, Jing Si, Bao-Kai Cui

**Affiliations:** 1 Institute of Microbiology, School of Ecology and Nature Conservation, Beijing Forestry University, Beijing 100083, China Beijing Forestry University Beijing China; 2 Biotechnology Research Institute, Xinjiang Academy of Agricultural and Reclamation Sciences / Xinjiang Production & Construction Group Key Laboratory of Crop Germplasm Enhancement and Gene Resources Utilization, Shihezi, Xinjiang 832000, China Biotechnology Research Institute, Xinjiang Academy of Agricultural and Reclamation Sciences Shihezi China

**Keywords:** core polyporoid clade, molecular phylogeny, polypore, taxonomy, wood-decaying fungi

## Abstract

A new poroid wood-inhabiting fungal genus, *Crassisporus* gen. nov., is proposed on the basis of morphological characters and molecular evidence. The genus is characterized by an annual growth habit, effused-reflexed to pileate basidiocarps with pale yellowish brown to yellowish brown, concentrically zonate or sulcate, and velutinate pileal surface, a trimitic hyphal system with clamped generative hyphae, tissues turning to dark in KOH, oblong to broadly ellipsoid, hyaline, smooth, and slightly thick-walled basidiospores. Phylogenetic analysis based on ITS+nLSU sequences indicate that *Crassisporus* belongs to the core polyporoid clade. The combined ITS+nLSU+mtSSU+EF1-α+RPB2 sequences dataset of representative taxa in the Polyporaceae demonstrate that *Crassisporus* is grouped with *Haploporus* but forms a monophyletic lineage. In addition, four new species of *Crassisporus*, *C. imbricatus*, *C. leucoporus*, *C. macroporus*, and *C. microsporus* are described.

## Introduction

Polyporales is one of the most diverse orders of Basidiomycota including more than 1800 described species in 216 genera and 13 families (Kirk et al. 2008). In the last 10 years, many new genera in Polyporales have been established, such as *Datroniella* B.K. Cui, Hai J. Li & Y.C. Dai (Li et al. 2014a), *Fragifomes* B.K. Cui, M.L. Han & Y.C. Dai (Han et al. 2016), *Megasporia* B.K. Cui, Y.C. Dai & Hai J. Li (Li and Cui 2013), *Pseudofibroporia* Yuan Y. Chen & B.K. Cui (Chen et al. 2017), and *Pseudomegasporoporia* X.H.Ji & F.Wu (Ji and Wu 2017).

During the investigations of species diversity and phylogeny of Polyporales, four new species were found that did not belong to any known genus, for which reason, a new genus is established to accommodate them. Morphologically, these four taxa do not fit any of the known polypore taxa. To confirm the position of the new genus, phylogenetic analyses of the new genus and related taxa within Polyporales were carried out based on the internal transcribed spacer (ITS) regions, the large subunit nuclear ribosomal RNA gene (nLSU), the small subunit mitochondrial rRNA gene sequences (mtSSU), the translation elongation factor 1-α gene (EF1-α), and the second largest subunit of RNA polymerase II (RPB2).

## Materials and methods

### Morphological studies

The studied specimens were deposited at the herbarium of the Institute of Microbiology, Beijing Forestry University (BJFC). Macro-morphological descriptions are based on the field notes and measurements of herbarium specimens. Color terms follow Petersen (1996). Micro-morphological data were obtained from the dried specimens and observed under a light microscope following Shen et al. (2019). Sections were studied at magnifications up to 1000× using a Nikon E80i microscope and phase contrast illumination (Nikon, Tokyo, Japan). Drawings were made with the aid of a drawing tube. Microscopic features, measurements, and drawings were made from slide preparations stained with cotton blue and Melzer’s reagent. Spores were measured from sections cut from the tubes. In presenting the variation of spore size, 5% of measurements were excluded from each end of the range, and were given in parentheses. The following abbreviations were used: **KOH**, 5% potassium hydroxide; **IKI**, Melzer’s reagent; **IKI-**, neither amyloid nor dextrinoid; **CB**, cotton blue; **CB+**, cyanophilous; **CB-**, acyanophilous; **L**, mean spore length (arithmetic average of all spores); **W**, mean spore width (arithmetic average of all spores); **Q**, variation in the L/W ratios between the specimens studied; ***n*** (a/b), number of spores (a) measured from given number (b) of specimens.

### DNA extraction and sequencing

A CTAB rapid plant genome extraction kit (Aidlab Biotechnologies Co. Ltd, Beijing) was used to extract total genomic DNA from dried specimens, and performed the polymerase chain reaction (**PCR**) according to the manufacturer’s instructions with some modifications (Chen et al. 2015). The ITS region was amplified with primer pairs ITS5 and ITS4 (White et al. 1990). The nLSU region was amplified with primer pairs LR0R and LR7 (http://www.biology.duke.edu/fungi/mycolab/primers.htm). The mtSSU region was amplified with primer pairs MS1 and MS2 (White et al. 1990). Part of EF1-α was amplified with primer pairs EF1-983F and EF1-1567R (Rehner and Buckley 2005). RPB2 was amplified with primer pairs fRPB2-5F and fRPB2-7cR or bRPB2-6F and bRPB2-7R (Liu et al. 1999; Matheny 2005). The PCR procedure for ITS, mtSSU and EF1-α was as follows: initial denaturation at 95 °C for 3 min, followed by 34 cycles at 94 °C for 40 s, 54 °C for ITS and mtSSU, 54–56 °C for EF1-α for 45 s, 72 °C for 1 min, and a final extension of 72 °C for 10 min. The PCR procedure for nLSU was as follows: initial denaturation at 94 °C for 1 min, followed by 34 cycles at 94 °C for 30 s, 50 °C for 1 min, 72 °C for 1.5 min, and a final extension of 72 °C for 10 min. The PCR procedure for RPB2 was as follows: initial denaturation at 94 °C for 2 min, followed by 10 cycles at 94 °C for 40 s, 60 °C for 40 s and 72 °C for 2 min, then followed by 37 cycles at 94 °C for 45 s, 55–57 °C for 1.5 min and 72 °C for 2 min, and a final extension of 72 °C for 10 min. The PCR products were purified and sequenced at Beijing Genomics Institute, China, with the same primers. All newly generated sequences were submitted to GenBank (Table 1).

**Table 1.** Species, specimens and GenBank accession numbers of sequences used in this study.

Species	Sample no.	Locality	GenBank accessions				
			ITS	nLSU	mtSSU	EF1-α	RPB2
*Abortiporus biennis*	EL 65-03	Sweden	JN649325	JN649325	–	–	–
*Abundisporus fuscopurpureus*	Cui 10950	China	KC456254	KC456256	KF051025	KF181154	–
*A. pubertatis*	Dai 11310	China	KC787568	KC787575	KF051031	KF181125	
	Dai 11927	China	KC787569	KC787576	KF051034	KF181128	–
*A. sclerosetosus*	MUCL 41438	Singapore	FJ411101	FJ393868	–	–	–
*A. violaceus*	Ryvarden 32807	Finland	KF018127	KF018135	KF051038	KF181132	–
*Antrodia albida*	CBS 308.82	USA	DQ491414	AY515348	–	–	–
*A. macra*	MUAF 887	Czech Republic	EU340898	–	–	–	–
*Bjerkandera adusta*	NBRC 4983	Unknown	AB733156	AB733333	–	–	–
*Cinereomyces lindbladii*	KHL 12078	Norway	FN907906	FN907906	–	–	–
*Climacocystis borealis*	KHL 13318	Estonia	JQ031126	JQ031126	–	–	–
*Coriolopsis brunneoleuca*	Cui 13911	China	MK116480 ^a^	MK116489 ^a^	MK116498 ^a^	–	MK124544 ^a^
*C. brunneoleuca*	Dai 12180	China	KC867414	KC867432	–	–	KF274655
*C. polyzona*	BKW004	Ghana	JN164978	JN164790	–	JN164881	JN164856
*C. retropicta*	Cui 13849	China	MK116481 ^a^	MK116490 ^a^	MK116499 ^a^	MK122979 ^a^	MK124545 ^a^
	Cui 14030	China	MK116482 ^a^	MK116491 ^a^	MK116500 ^a^	MK122980 ^a^	MK122987 ^a^
*Crassisporus imbricatus*	Cui 6556	China	KC867351	KC867426	–	–	–
*C. imbricatus*	Dai 10788	China	KC867350	KC867425	KX838374	–	–
*C. leucoporus*	Cui 16801	Australia	MK116488 ^a^	MK116497 ^a^	MK116507 ^a^	MK122986 ^a^	MK122993 ^a^
*C. macroporus*	Cui 14465	China	MK116485 ^a^	MK116494 ^a^	MK116504 ^a^	MK122983 ^a^	MK122990 ^a^
	Cui 14468	China	MK116486 ^a^	MK116495 ^a^	MK116505 ^a^	MK122984 ^a^	MK122991 ^a^
*C. microsporus*	Cui 16221	China	MK116487 ^a^	MK116496 ^a^	MK116506 ^a^	MK122985 ^a^	MK122992 ^a^
*Daedaleopsis confragosa*	Cui 6892	China	KU892428	KU892448	KX838381	KX838418	KU892507
*D. hainanensis*	Cui 5178	China	KU892435	KU892462	KX838413	KX838441	KU892495
*Datronia mollis*	RLG6304sp	USA	JN165002	JN164791	–	JN164901	JN164872
*Earliella scabrosa*	PR1209	Puerto Rico	JN165009	JN164793	–	–	JN164866
*Fomes fomentarius*	ES 2008-3	Sweden	JX109860	JX109860	–	–	–
*Fomitella supina*	JV0610	Guatemala	KF274645	KF274646	–	–	–
*F. supina*	Ryvarden 39027	Puerto Rico	KF274643	–	–	–	–
*Fomitopsis betulina*	Dai 11449	China	KR605798	KR605737	KR605998	KR610726	KR610816
*F. pinicola*	Cui 10405	China	KC844852	KC844857	KR605961	KR610690	KR610781
*Fragiliporia fragilis*	Dai 13080	China	KJ734260	KJ734264	–	–	–
*F. fragilis*	Dai 13559	China	KJ734261	KJ734265	–	–	–
	Yuan 5516	China	KJ734263	KJ734267	–	–	–
*Funalia gallica*	Dai 10977	China	KC867378	KC867452	–	–	KU182651
*F. trogii*	RLG4286Sp	USA	JN164993	JN164808	–	JN164898	JN164867
*Gelatoporia subvermispora*	BRNU 592909	Czech Republic	FJ496694	FJ496706	–	–	–
*Grammethelopsis subtropica*	Cui 9035	China	JQ845094	JQ845097	KF051030	KF181124	–
	Cui 9041	China	JQ845096	JQ845099	KF051039	KF181133	–
*Haploporus latisporus*	Dai 11873	China	KU941847	KU941871	KU941896	KU941934	KU941918
*H. latisporus*	Dai 10562	China	KU941848	KU941872	KU941897	KU941935	KU941919
*H. odorus*	Yuan 2365	China	KU941846	KU941870	KU941895	KU941933	KU941917
	Dai 11296	China	KU941845	KU941869	KU941894	KU941932	KU941916
*H. subtrameteus*	Dai 4222	China	KU941849	KU941873	KU941898	KU941936	KU941920
	Cui 10656	China	KU941850	KU941874	KU941899	KU941937	KU941921
*Heterobasidion annosum*	PFC 5327	Greece	KC492915	–	–	KC571655	–
*Hexagonia apiaria*	Cui 6447	China	KC867362	KC867481	MG847228	MG867697	KF274660
*H. apiaria*	Dai 10784	China	KX900635	KX900682	KX900732	KX900822	MG867677
*H. hirta*	Dai 5081	China	–	KC867486	–	–	–
	Cui 4051	China	KC867359	KC867471	–	–	–
*Hornodermoporus latissimus*	Cui 6625	China	HQ876604	JF706340	KF051040	KF181134	–
	Dai 12054	China	KX900639	KX900686	KF218297	KF286303	–
*H. martius*	MUCL 41677	Argentina	FJ411092	FJ393859	–	–	–
	MUCL 41678	Argentina	FJ411093	FJ393860	–	–	–
*Hydnopolyporus fimbriatus*	LR 40855	Puerto Rico	JN649347	JN649347	–	–	–
*Hypochnicium lyndoniae*	NL 041031	UK	JX124704	JX124704	–	–	–
*Laetiporus montanus*	Cui 10011	China	KF951274	KF951315	KX354570	KX354617	KT894790
*L. sulphureus*	Cui 12388	China	KR187105	KX354486	KX354560	KX354607	KX354652
*Lenzites betulina*	HHB9942Sp	USA	JN164983	JN164794	–	JN164895	JN164860
*Megasporia ellipsoidea*	Cui 13854	China	MK116483 ^a^	MK116492 ^a^	MK116501 ^a^	MK122981 ^a^	MK122988 ^a^
*M. major*	Cui 10253	China	JQ314366	JQ780437	MK116502 ^a^	–	–
*Megasporoporiella rhododendri*	Cui 10745	China	MK116484 ^a^	MK116493 ^a^	MK116503 ^a^	MK12298222982^a^	MK122989 ^a^
*M. subcavernulosa*	Cui 14247	China	MG847213	MG847222	MG847234	MG867705	MG867685
*Microporus affinis*	Cui 7714	China	JX569739	JX569746	KX880696	–	KF274661
*M. vernicipes*	Dai 9283	China	KX880618	KX880658	KX880701	KX880926	–
*M. xanthopus*	Cui 8284	China	JX290074	JX290071	KX880703	KX880878	JX559313
*Neodatronia sinensis*	Dai 11921	China	JX559272	JX559283	–	–	JX559320
*Neofomitella fumosipora*	Cui 8816	China	JX569734	JX569741	KX900766	–	–
	Cui 13581a	China	KX900664	KX900714	KX900767	KX900848	KX900815
*N. rhodophaea*	TFRI 414	Unknown	EU232216	EU232300	–	–	–
*Obba rivulosa*	KCTC 6892	Canada	FJ496693	FJ496710	–	–	–
*Perenniporia hainaniana*	Cui 6364	China	JQ861743	JQ861759	KF051044	KF181138	–
*P. hainaniana*	Cui 6365	China	JQ861744	JQ861760	KF051045	KF181139	–
*P. medulla-panis*	MUCL 49581	Poland	FJ411088	FJ393876	–	–	–
	Cui 14515	China	MG847214	MG847223	–	MG867707	MG867687
*P. substraminea*	Cui 10177	China	JQ001852	JQ001844	KF051046	KF181140	–
	Cui 10191	China	JQ001853	JQ001845	KF051047	KF181141	–
*Perenniporiella chaquenia*	MUCL 47648	Argentina	FJ411084	FJ393856	–	HM467610	–
*P. micropora*	MUCL 43581	Cuba	FJ411086	FJ393858	–	HM467608	–
*P. neofulva*	MUCL 45091	Cuba	FJ411080	FJ393852	–	HM467599	–
*P. pendula*	MUCL 46034	Cuba	FJ411081	FJ393853	–	HM467601	–
*Phanerochaete chrysosporium*	BKM-F-1767	USSR	HQ188436	GQ470643	–	–	–
*Phlebia unica*	KHL 11786	Sweden	EU118657	EU118657	–	–	–
*Pycnoporus cinnabarinus*	Dai 14386	China	KX880629	KX880667	KX880712	KX880885	KX880854
*Skeletocutis amorpha*	Miettinen 11038	Finland	FN907913	FN907913	–	–	–
*Stereum hirsutum*	NBRC 6520	Unknown	AB733150	AB733325	–	–	–
*Trametes conchifer*	FP106793Sp	USA	JN164924	JN164797	–	JN164887	JN164849
*T. pubescens*	FP101414Sp	USA	JN164963	JN164811	–	JN164889	JN164851
*T. tephroleuca*	Cui 7987	China	KC848293	KC848378	KX880755	KX880934	KX880869
*T. versicolor*	FP135156Sp	USA	JN164919	JN164809	–	JN164878	JN164850
*Truncospora detrita*	MUCL 42649	French Guyana	FJ411099	FJ393866	–	–	–
*T. macrospora*	Cui 8106	China	JX941573	JX941596	KX880763	KX880920	KX880871
	Yuan 3777	China	JX941574	JX941597	–	–	–
*T. ochroleuca*	MUCL 39726	China Taiwan	FJ411098	FJ393865	–	–	–
	Cui 5671	China	JX941584	JX941602	KF218309	KF286315	–
*T. ohiensis*	MUCL 41036	USA	FJ411096	FJ393863	–	–	–
*Tyromyces chioneus*	Cui 10225	China	KF698756	KF698745	–	–	–
*T. kmetii*	Penttilä 13474	China	KF705040	KF705041	–	–	–
*Vanderbylia fraxinea*	DP 83	Italy	AM269789	AM269853	–	–	–
	MUCL 39326	France	FJ411094	FJ393861	–	–	–
*V. robiniophila*	Cui 5644	China	HQ876609	JF706342	KF051051	KF181145	MG867691
	Cui 7144	China	HQ876608	JF706341	KF051052	KF181146	–
*V. vicina*	MUCL 44779	Ethiopia	FJ411095	FJ393862	–	–	–
*Whitfordia scopulosa*	Dai 10739	China	KC867364	KC867482	KX880766	KX880922	MG867692

^a^ Newly generated sequences for this study

### Phylogenetic analyses

Sequences used for phylogenetic analyses in this study are listed in Table 1. Sequences of ITS, nLSU, mtSSU, EF1-α, and RPB2 were aligned initially in MAFFT 7 (Katoh and Standley 2013; http://mafft.cbrc.jp/alignment/server/) and then manually adjusted in BioEdit (Hall 1999). Finally, these gene fragments were concatenated with Mesquite 3.2 (Maddison and Maddison 2017) for further phylogenetic analyses. Phylogenies were inferred from the combined 2-gene dataset (ITS+nLSU) and 5-gene dataset (ITS+nLSU+mtSSU+EF1-α+RPB2). *Heterobasidion annosum* (Fr.) Bref. and *Stereum hirsutum* (Willd.) Pers. obtained from GenBank were used as outgroups to root trees in the 2-gene based analysis. *Laetiporus montanus* Černý ex Tomšovský & Jankovský and *L. sulphureus* (Bull.) Murrill were selected as outgroups to root trees in the 5-gene based analysis. The final concatenated sequence alignments were deposited in TreeBase (https://treebase.org/treebase-web/home.html; submission ID 23521).

Phylogenetic analyses used in this study followed the approach of Zhu et al. (2019) and Song and Cui (2017). Maximum parsimony (**MP**) analysis was performed in PAUP* v. 4.0b10 (Swofford 2002). All characters were equally weighted and gaps were treated as missing data. Trees were inferred using the heuristic search option with TBR branch swapping and 1000 random sequence additions. Max-trees were set to 5000, branches of zero length were collapsed, and all parsimonious trees were saved. Clade robustness was assessed using a bootstrap (**BT**) analysis with 1000 replicates (Felsenstein 1985). Descriptive tree statistics tree length (**TL**), consistency index (**CI**), retention index (**RI**), rescaled consistency index (**RC**) and homoplasy index (**HI**) were calculated for each maximum parsimonious tree generated.

RAxML v. 7.2.6 (Stamatakis 2006) was used to perform maximum likelihood (ML) analysis involved 200 ML searches under the GTR+GAMMA model and only the best tree from all searches was kept. In addition, 200 rapid bootstrap replicates were run with the GTR+CAT model to assess the reliability of the nodes.

MrModeltest v. 2.3 (Posada and Crandall 1998; Nylander 2004) was used to determine the best fit evolution model for the combined multi-gene dataset for Bayesian inference (BI). Bayesian inference was calculated with MrBayes v. 3.1.2 with a general time reversible (GTR) model of DNA substitution and a gamma distribution rate variation across sites (Ronquist and Huelsenbeck 2003). Four Markov chains were run for two runs from random starting trees for 2 million generations (ITS+nLSU), for 5 million generations (ITS+nLSU+mtSSU+EF1-α+RPB2) until the split deviation frequency value <0.01, and trees were sampled every 100 generation. The first quarter generations were discarded as burn-in. A majority rule consensus tree of all remaining trees was calculated.

Phylogenetic trees were viewed using FigTree v. 1.4.2 (http://tree.bio.ed.ac.uk/software/figtree/). Branches that received bootstrap support for maximum parsimony (MP), maximum likelihood (ML) and Bayesian posterior probabilities (BPP) greater than or equal to 75% (MP and ML) and 0.95 (BPP) were considered as significantly supported, respectively.

## Results

### Molecular phylogeny

**Figure 1.**
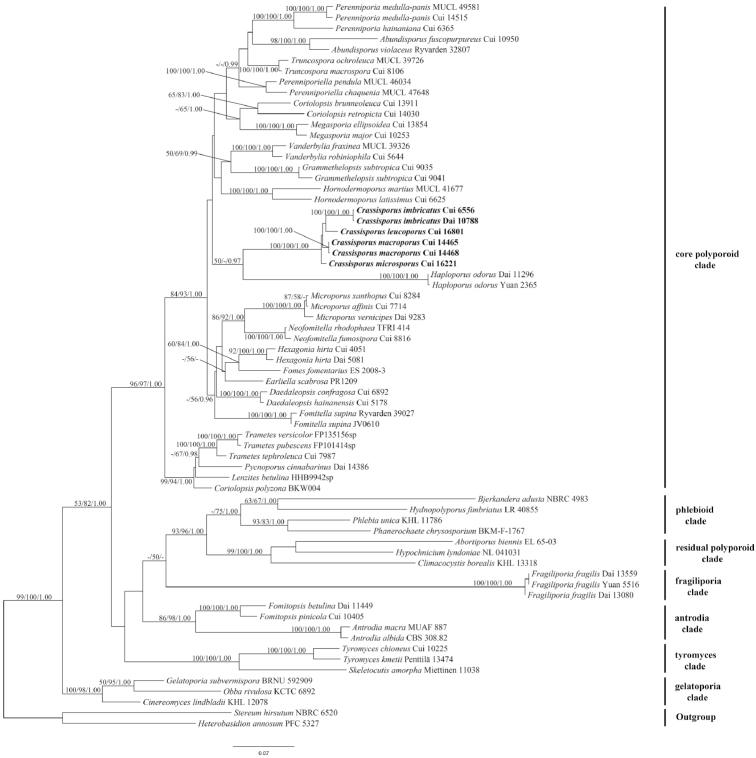
Phylogeny of *Crassisporus* and related genera in Polyporales based on combined ITS and nLSU sequences. Topology is from ML analysis with parsimony bootstrap support values (≥50 %), maximum likelihood bootstrap support values (≥50 %) and Bayesian posterior probability values (≥0.95).

The combined 2-gene dataset included sequences from 68 fungal samples representing 59 taxa. The dataset had an aligned length of 2111 characters, of which 1249 characters were constant, 196 were variable and parsimony-uninformative, and 666 were parsimony-informative. MP analysis yielded 37 equally parsimonious trees (TL = 4143, CI = 0.345, RI = 0.617, RC = 0.213, HI = 0.655). Best model for the combined 2-gene dataset estimated and applied in the BI was GTR+I+G, lset nst = 6, rates = invgamma; prset statefreqpr = dirichlet (1,1,1,1). MP, ML and BI analyses yielded similar tree topologies with an average standard deviation of split frequencies = 0.006293 (BI), and the ML topology is shown in Figure 1. The phylogeny (Fig. 1) inferred from the combined ITS+nLSU sequences demonstrated seven major clades for 59 species of the Polyporales. The new genus *Crassisporus* embed in the core polyporoid clade and grouped with *Haploporus* Bondartsev & Singer.

**Figure 2.**
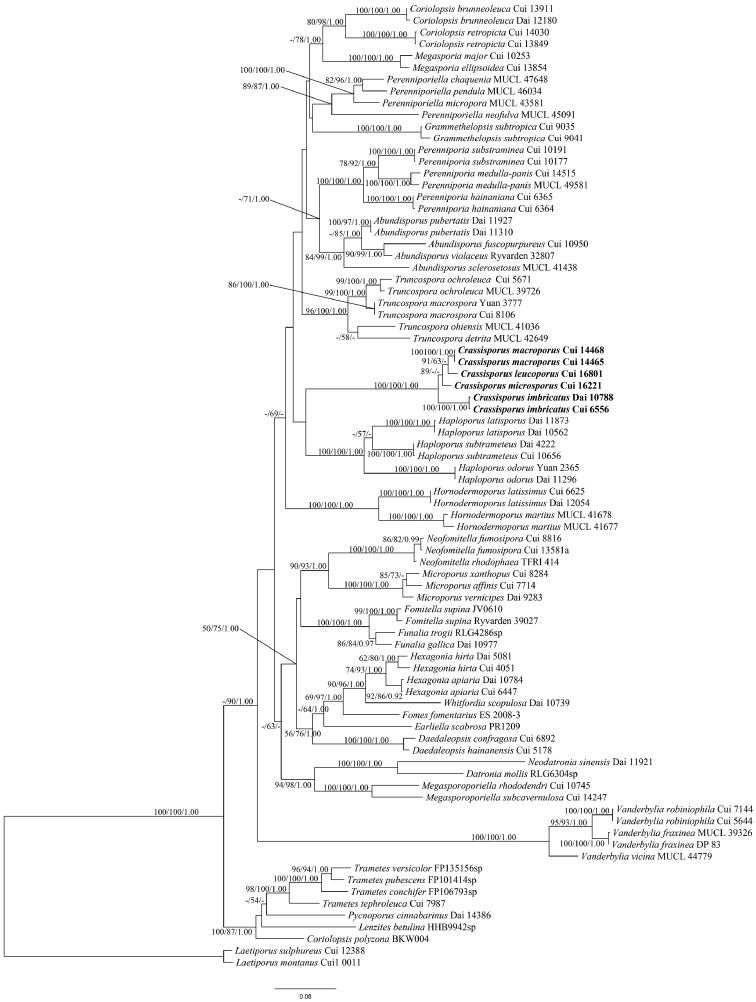
Phylogeny of *Crassisporus* and related species obtained for more representative taxa in the Polyporaceae based on combined sequences dataset of ITS+nLSU+mtSSU+EF1-α+RPB2. Topology is from ML analysis with parsimony bootstrap support values (≥50 %), maximum likelihood bootstrap support values (≥50 %), and Bayesian posterior probability values (≥0.95).

The combined 5-gene (ITS, nLSU, mtSSU, EF1-α, RPB2) dataset included sequences of 82 fungal samples representing 57 taxa. The dataset had an aligned length of 4306 characters, of which 2521 characters were constant, 258 were variable and parsimony-uninformative, and 1527 were parsimony-informative. MP analysis yielded 1 equally parsimonious tree (TL = 8989, CI = 0.339, RI = 0.620, RC = 0.210, HI = 0.661). Bayesian and ML analyses resulted in a similar topology as the MP analysis, with an average standard deviation of split frequencies = 0.006328 (BI); and the ML topology is shown in Figure 2. A further phylogeny (Fig. 2) inferred from the combined 5-gene dataset was obtained for more representative taxa in the Polyporaceae and showed that the new genus grouped with *Haploporus* clade but distinctly formed a monophyletic lineage.

## Taxonomy

### Crassisporus

Taxon classificationFungiPolyporalesPolyporaceae

B.K. Cui & Xing Ji gen. nov.

B960EFA709CE585D9A1A3C7143619ACB

828486

#### Notes.

Differs from other genera by the combination of effused-reflexed to pileate basidiocarps, pale yellowish brown to yellowish brown, concentrically zonate or sulcate, velutinate pileal surface, a trimitic hyphal system with clamped generative hyphae, tissues darkening in KOH, and oblong to broadly ellipsoid, hyaline, smooth and slightly thick-walled basidiospores.

#### Etymology.

*Crassisporus* (Lat.): referring to thick-walled basidiospores.

#### Type species.

*Crassisporus macroporus* B.K. Cui & Xing Ji.

Basidiocarps annual, effused-reflexed to pileate. Pileal surface pale yellowish brown, yellowish brown to umber-brown when dry, concentrically zonate or sulcate, velutinate. Pore surface usually white, cream buff to cinnamon-buff when fresh, buff, pale yellowish brown to yellowish brown when dry. Context pale yellowish brown to yellowish brown, leathery to corky when dry. Tubes concolorous with the context, corky when dry. Hyphal system trimitic with clamped generative hyphae, skeletal hyphae hyaline to pale yellowish brown, binding hyphae hyaline to pale yellowish brown, negative in Melzer’s reagent, tissues turning to black in KOH. Cystidia absent, thin-walled cystidioles usually present. Basidiospores oblong to broadly ellipsoid, hyaline, smooth, slightly thick-walled, IKI-, CB-. Causing a white rot.

### Crassisporus imbricatus

Taxon classificationFungiPolyporalesPolyporaceae

B.K. Cui & Xing Ji sp. nov.

0553B393CCA55E658DE28ECE51DD0D6F

828487

Figs 3 , 4

#### Notes.

*Crassisporus imbricatus* is characterized by imbricate basidiocarps, pale greyish-brown pore surface when dry, round to angular pores (3–5 per mm), and oblong ellipsoid basidiospores (10–14 × 4.5–6.2 μm).

#### Holotype.

CHINA. Hainan Province, Changjiang County, Bawangling Nature Reserve, on dead angiosperm tree, 9 May 2009, Dai 10788 (BJFC).

#### Etymology.

*Imbricatus* (Lat.): referring to the imbricate basidiocarps.

#### Description.

Fruitbody: Basidiocarps annual, effused-reflexed to pileate, imbricate, soft corky, without odor or taste when fresh, leathery to corky upon drying. Pilei semicircular or elongated, projecting up to 1.5 cm, 3.5 cm wide, and 2.5 mm thick at base. Pileal surface yellowish brown, velutinate, concentrically zonate. Pore surface buff when fresh, becoming pale greyish brown when dry; sterile margin indistinct, pores round to angular, 3–5 per mm; dissepiments slightly thick, entire to slightly lacerate. Context yellowish brown, leathery, up to 2.5 mm thick. Tubes concolorous with context, corky, up to 1.5 mm long.

Hyphal structure: Hyphal system trimitic; generative hyphae bearing clamp connections; skeletal and binding hyphae IKI-, CB-; tissues turning to black in KOH.

Context: Generative hyphae infrequent, hyaline, thin-walled, unbranched, 2–3.5 μm in diam.; skeletal hyphae dominant, hyaline to pale yellowish brown, thick-walled with a narrow lumen to subsolid, rarely branched, straight, interwoven, occasionally simple-septate, 2.5–5.5 μm in diam.; binding hyphae hyaline to pale yellowish brown, thick-walled with a narrow lumen to subsolid, flexuous, frequently branched, interwoven, 1.2–2.5 μm in diam.

Tubes: Generative hyphae infrequent, hyaline, thin-walled, occasionally branched, 1.5–3 μm in diam.; skeletal hyphae dominant, hyaline to pale yellowish brown, thick-walled, occasionally branched, strongly interwoven, rarely simple-septate, 1.5–3.5 μm in diam.; binding hyphae hyaline to pale yellowish brown, thick-walled with a narrow lumen to subsolid, flexuous, frequently branched, interwoven, 1–2 μm in diam. Cystidia and cystidioles absent. Basidia clavate, bearing four sterigmata and a basal clamp connection, 19–32 × 9–12 μm; basidioles dominant, in shape similar to basidia, but distinctly smaller.

Spores: Basidiospores oblong ellipsoid, hyaline, slightly thick-walled, smooth, IKI-, CB-, 10–14(–15) × 4.5–6.2(–6.6) μm, L = 12.33 μm, W = 5.34 μm, Q = 2.27–2.36 (*n* = 60/2).

#### Type of rot.

White rot.

#### Additional specimen (paratype) examined.

CHINA. Hainan Province, Changjiang County, Bawangling Nature Reserve, on fallen branch of *Pinus latteri*, 10 May 2009, Cui 6556 (BJFC).

**Figure 3.**
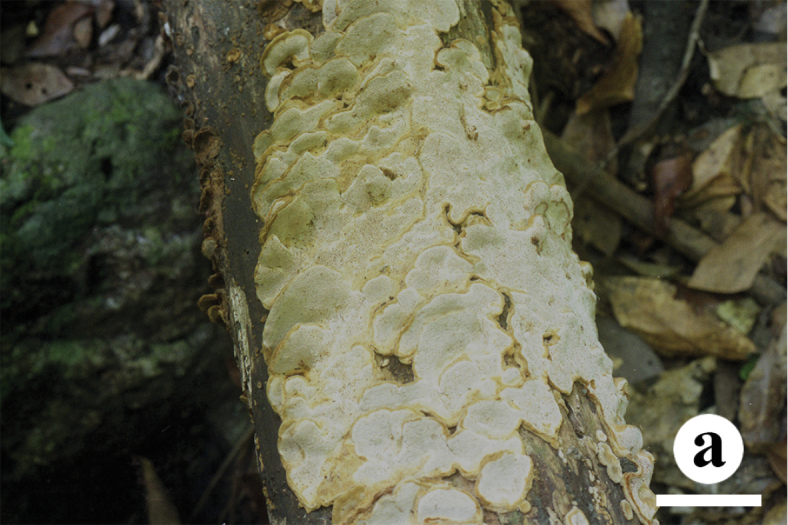
Basidiocarps of *Crassisporus imbricatus*. Scale bar: 2 cm.

**Figure 4.**
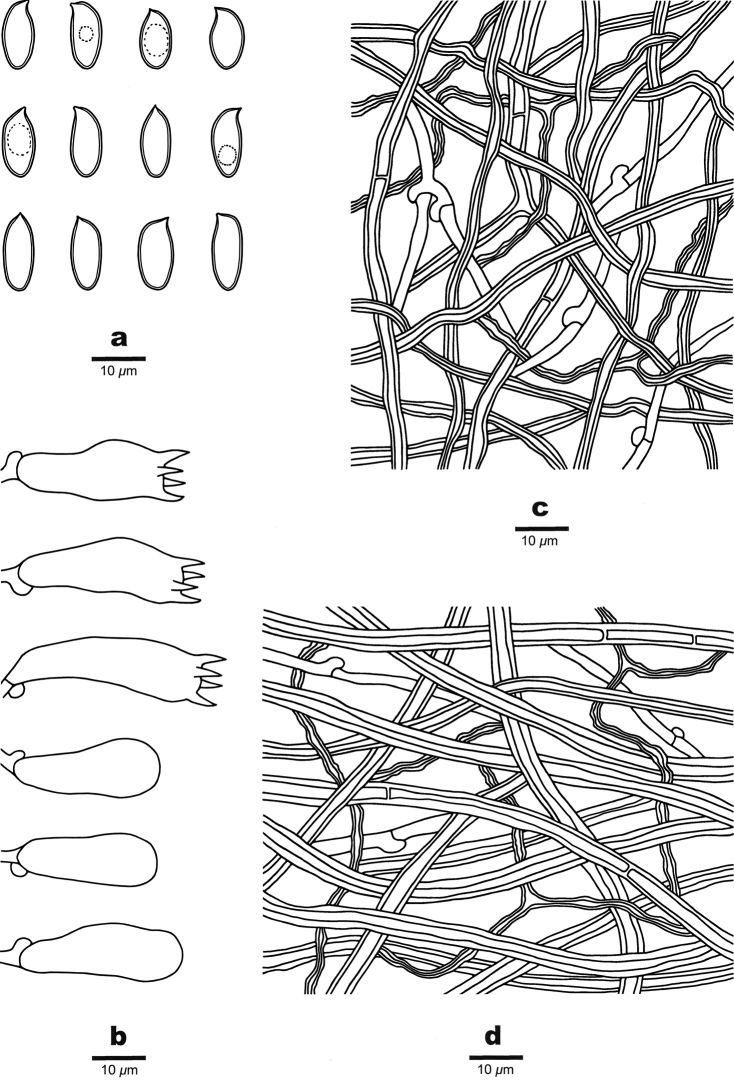
Microscopic structures of *Crassisporus imbricatus* (drawn from the holotype) **A** basidiospores **B** basidia and basidioles **C** hyphae from trama **D** hyphae from context.

### Crassisporus leucoporus

Taxon classificationFungiPolyporalesPolyporaceae

B.K. Cui & Xing Ji sp. nov.

D4DB2FEB8DF3520682423C6FAC93540B

828488

Figs 5 , 6

#### Notes.

*Crassisporus leucoporus* is characterized by a white pore surface when fresh, round to angular pores (3–4 per mm) and oblong ellipsoid basidiospores (8.4–11.2 × 4.2–5.4 μm).

#### Holotype.

AUSTRALIA. Queensland, Cairns, Roadside of Mount Whitfield Park, on fallen angiosperm branch, 18 May 2018, Cui 16801 (BJFC).

#### Etymology.

*Leucoporus* (Lat.): referring to the white pore surface when fresh.

#### Description.

Fruitbody: Basidiocarps annual, effused-reflexed to pileate, corky, without odor or taste when fresh, soft leathery to corky upon drying. Pilei semicircular or elongated, projecting up to 1.5 cm, 3 cm wide, and 6 mm thick at base. Pileal surface yellowish brown to umber-brown, finely velutinate, concentrically sulcate. Pore surface white when fresh, becoming cream, clay buff to pale yellowish brown when dry; sterile margin distinct, cream to pale yellowish brown, up to 1.5 mm wide; pores round to angular, 3–4 per mm; dissepiments slightly thick, entire. Context pale yellowish brown to fulvous, leathery, up to 3 mm thick. Tubes pale yellowish brown, corky, up to 2.5 mm long.

Hyphal structure: Hyphal system trimitic; generative hyphae bearing clamp connections; skeletal and binding hyphae IKI-, CB-; tissues turning to black in KOH.

Context: Generative hyphae infrequent, hyaline, thin-walled, unbranched, 1.1–2.6 μm in diam.; skeletal hyphae in context dominant, pale yellowish brown, thick-walled with a narrow to wide lumen, unbranched, straight, interwoven, occasionally simple-septate, 1.8–3.9 μm in diam.; binding hyphae hyaline to pale yellowish brown, thick-walled with a narrow lumen to subsolid, flexuous, frequently branched, interwoven, 0.7–2.2 μm in diam.

Tubes: Generative hyphae infrequent, hyaline, thin-walled, occasionally branched, 1–2.8 μm in diam.; skeletal hyphae dominant, hyaline to pale yellowish brown, thick-walled with a narrow to wide lumen, occasionally branched, more or less straight, strongly interwoven, 0.9–3.3 μm in diam.; binding hyphae hyaline to pale yellowish brown, thick-walled with a narrow lumen to subsolid, flexuous, frequently branched, interwoven, 0.8–2.1 μm in diam. Cystidia absent, cystidioles fusoid, sometimes septate at the tips, hyaline, thin-walled, 16.7–28.1 × 5.1–6.3 μm. Basidia clavate, bearing four sterigmata and a basal clamp connection, 18.1–29.2 × 6.4–9.8 μm; basidioles dominant, in shape similar to basidia, but smaller.

Spores: Basidiospores oblong ellipsoid, hyaline, smooth, slightly thick-walled, IKI-, CB-, (7.9–)8.4–11.2(–11.5) × (4–)4.2–5.4(–5.7) μm, L = 9.49 μm, W = 4.79 μm, Q = 1.99 (*n* = 60/1).

#### Type of rot.

White rot.

**Figure 5.**
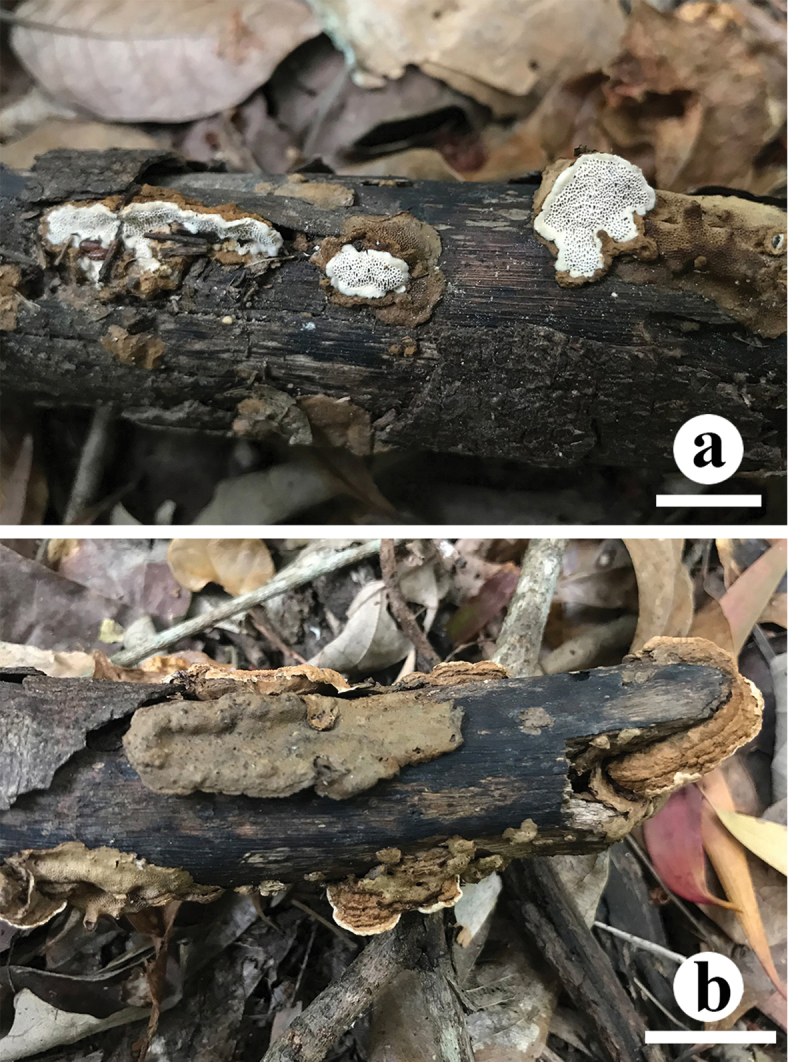
Basidiocarps of *Crassisporus leucoporus*. Scale bars: 1 cm (**A**); 2 cm (**B**).

**Figure 6.**
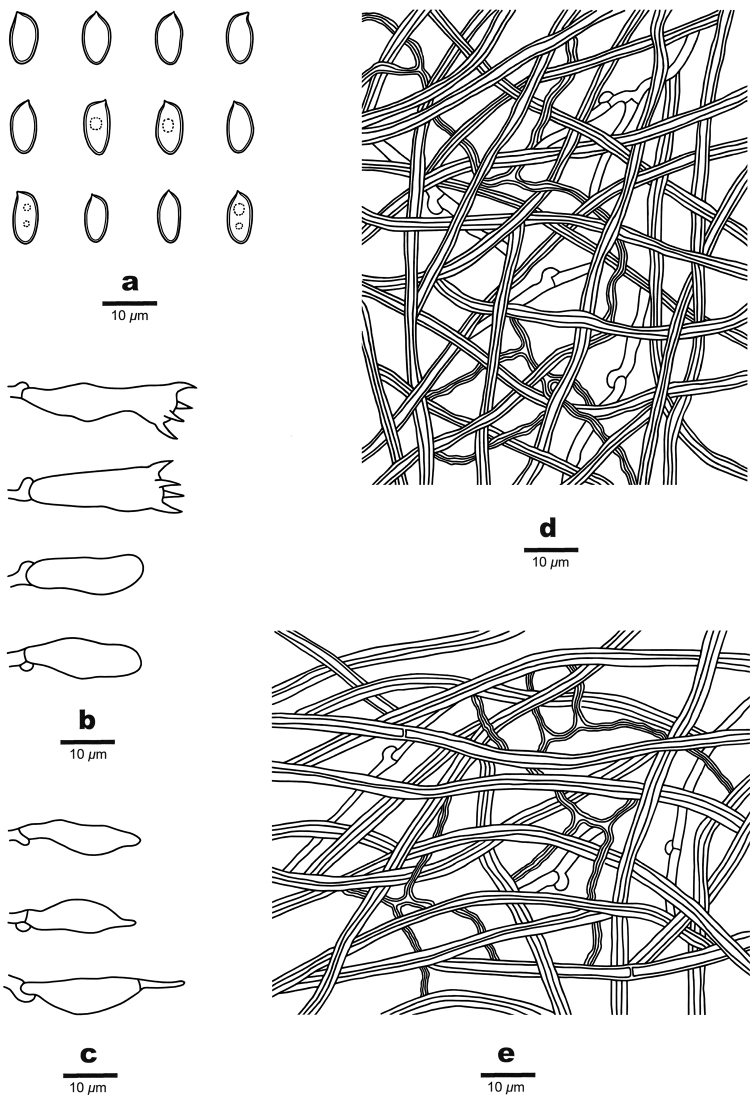
Microscopic structures of *Crassisporus leucoporus* (drawn from the holotype) **A** basidiospores **B** basidia and basidioles **C** cystidioles **D** hyphae from trama **E** hyphae from context.

### Crassisporus macroporus

Taxon classificationFungiPolyporalesPolyporaceae

B.K. Cui & Xing Ji sp. nov.

5A4BB7711CD1574BA88A00B6D8089E07

828489

Figs 7 , 8

#### Notes.

*Crassisporus macroporus* is characterized by cream-buff to cinnamon-buff colored pore surface with distinct sterile margin when fresh, large pores (2–3 per mm) with thin dissepiments, a trimitic hyphal system with cyanophilous skeletal hyphae, the presence of fusoid cystidioles, and oblong ellipsoid basidiospores (9.5–13.2 × 4–6.2 μm).

#### Holotype.

CHINA. Guangxi Autonomous Region, Huanjiang County, Mulun Nature Reserve, on fallen angiosperm branch, 10 July 2017, Cui 14468 (BJFC).

#### Etymology.

*Macroporus* (Lat.): referring to the large pores.

#### Description.

Fruitbody: Basidiocarps annual, effused-reflexed to pileate, corky to leathery, without odor or taste when fresh, soft leathery upon drying. Pilei flabelliform, semicircular or elongated, projecting up to 1.5 cm, 4 cm wide and 5 mm thick at base; resupinate part up to 7 cm long, 4 cm wide, and 5 mm thick at center. Pileal surface buff to yellowish brown when fresh, becoming yellowish brown upon drying, finely velutinate, concentrically sulcate. Pore surface cream, buff to cinnamon-buff when fresh, becoming buff, pale yellowish brown to yellowish brown when dry; sterile margin distinct, buff to pale yellowish brown, up to 2 mm wide; pores round to angular, 2–3 per mm; dissepiments thin, entire to lacerate. Context yellowish brown to pale yellowish brown, leathery, up to 1.5 mm thick. Tubes pale yellowish brown, corky, up to 2 mm long.

Hyphal structure: Hyphal system trimitic; generative hyphae bearing clamp connections; skeletal and binding hyphae IKI-, CB+; tissues turning to black in KOH.

Context: Generative hyphae infrequent, hyaline, thin-walled, unbranched, 1.5–3.5 μm in diam.; skeletal hyphae dominant, pale yellowish brown, thick-walled with a narrow lumen to subsolid, unbranched, more or less straight, interwoven, occasionally simple-septate, 2–5.5 μm in diam.; binding hyphae hyaline to pale yellowish brown, thick-walled with a narrow lumen to subsolid, flexuous, frequently branched, interwoven, 1–3 μm in diam.

Tubes: Generative hyphae infrequent, hyaline, thin-walled, occasionally branched, 1–2 μm in diam.; skeletal hyphae dominant, hyaline to pale yellowish brown, thick-walled with a narrow lumen to subsolid, occasionally branched, more or less straight, strongly interwoven, 1.5–3 μm in diam.; binding hyphae hyaline to pale yellowish brown, thick-walled with a narrow lumen to subsolid, flexuous, frequently branched, interwoven, 0.8–2 μm in diam. Cystidia absent, cystidioles fusoid, hyaline, thin-walled, 13–20 × 4.5–6 μm. Basidia clavate, bearing four sterigmata and a basal clamp connection, 17–28 × 7–8 μm; basidioles dominant, in shape similar to basidia, but smaller.

Spores: Basidiospores oblong ellipsoid, hyaline, smooth, slightly thick-walled, IKI-, CB-, 9.5–13.2(–14) × 4–6.2(–6.5) μm, L = 11.24 μm, W = 4.96 μm, Q = 2.26–2.31 (*n* = 60/2).

#### Type of rot.

White rot.

#### Additional specimen (paratype) examined.

CHINA. Guangxi Autonomous Region, Huanjiang County, Mulun Nature Reserve, on dead angiosperm tree, 10 July 2017, Cui 14465 (BJFC).

**Figure 7.**
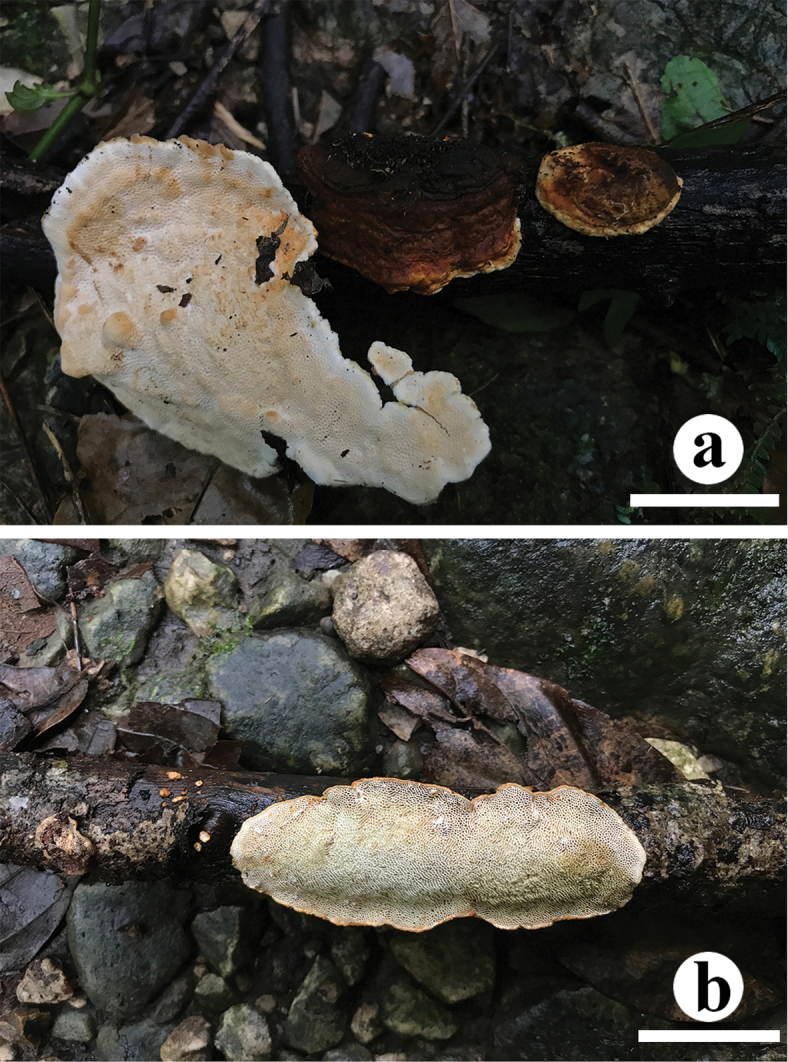
Basidiocarps of *Crassisporus macroporus*. Scale bars: 2 cm.

**Figure 8.**
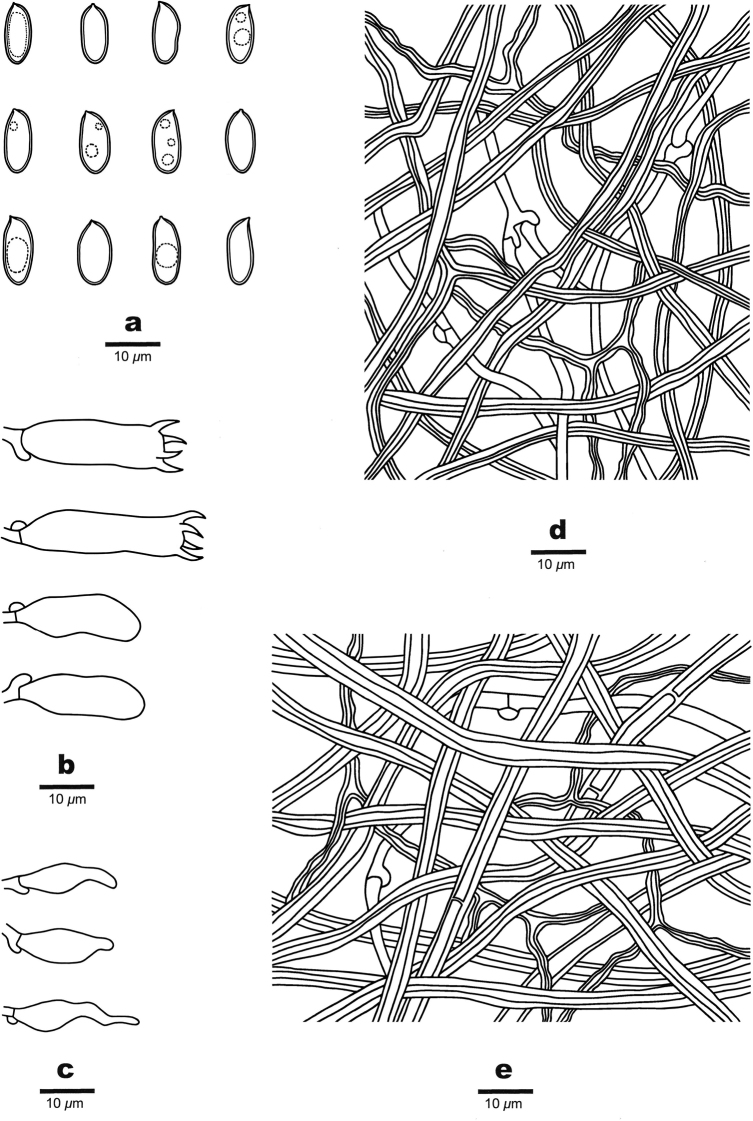
Microscopic structures of *Crassisporus macroporus* (drawn from the holotype) **A** basidiospores **B** basidia and basidioles **C** cystidioles **D** hyphae from trama **e** hyphae from context.

### Crassisporus microsporus

Taxon classificationFungiPolyporalesPolyporaceae

B.K. Cui & Xing Ji sp. nov.

DEEAF27C7EED5ADBB62D47DB3B190481

828514

Figs 9 , 10

#### Notes.

*Crassisporus microsporus* is characterized by pileate basidiocarps, small pores (5–7 per mm), and small, broadly ellipsoid basidiospores (4–5 × 3–3.7 μm).

#### Holotype.

CHINA. Yunnan Province, Ruili, Mori Tropical Rainforest Park, on living angiosperm tree, 17 September 2017, Cui 16221 (BJFC).

#### Etymology.

*Microsporus* (Lat.): referring to the small basidiospores.

#### Description.

Fruitbody: Basidiocarps annual, pileate, sessile, corky, without odor or taste when fresh, soft leathery to corky upon drying. Pilei semicircular, projecting up to 2 cm, 4 cm wide, and 4.5 mm thick at base. Pileal surface pale yellowish brown to yellowish brown, finely velutinate, concentrically sulcate. Pore surface cream, buff to cinnamon-buff when fresh, buff, pale yellowish brown to yellowish brown when dry; sterile margin distinct, buff, up to 1 mm wide; pores round to angular, 5–7 per mm; dissepiments slightly thick, entire. Context pale yellowish brown to yellowish brown, leathery to corky when dry, up to 1.5 mm thick. Tubes concolorous with context, soft corky to corky, up to 3 mm long.

Hyphal structure: Hyphal system trimitic; generative hyphae bearing clamp connections; skeletal and binding hyphae IKI-, CB-; tissues turning to deep brown in KOH.

Context: Generative hyphae infrequent, hyaline, thin-walled, occasionally branched, 1.2–3.5 μm in diam.; skeletal hyphae dominant, hyaline to pale yellowish brown, thick-walled with a narrow lumen, rarely branched, straight, interwoven, occasionally simple-septate, 2.5–6 μm in diam.; binding hyphae hyaline to pale yellowish brown, thick-walled with a narrow lumen to subsolid, flexuous, frequently branched, interwoven, 0.8–2.5 μm in diam.

Tubes: Generative hyphae infrequent, hyaline, thin-walled, rarely branched, 1.2–3 μm in diam.; skeletal hyphae dominant, hyaline to pale yellowish brown, thick-walled with a narrow lumen to subsolid, moderately branched, more or less straight, strongly interwoven, 1.5–3 μm in diam.; binding hyphae hyaline to pale yellowish brown, thick-walled with a narrow lumen to subsolid, flexuous, frequently branched, interwoven, 0.8–2.5 μm in diam. Cystidia absent, cystidioles fusoid, hyaline, thin-walled, 12.5–18 × 4–5.5 μm. Basidia clavate, bearing four sterigmata and a basal clamp connection, 14–21 × 4.5–6 μm; basidioles in shape similar to basidia, but distinctly smaller.

Spores: Basidiospores broadly ellipsoid, hyaline, smooth, slightly thick-walled, IKI-, CB-, 4–5(−5.2) × (−2.8)3–3.7(−3.9) μm, L = 4.5 μm, W =3.23 μm, Q = 1.4 (*n* = 60/1).

#### Type of rot.

White rot.

**Figure 9.**
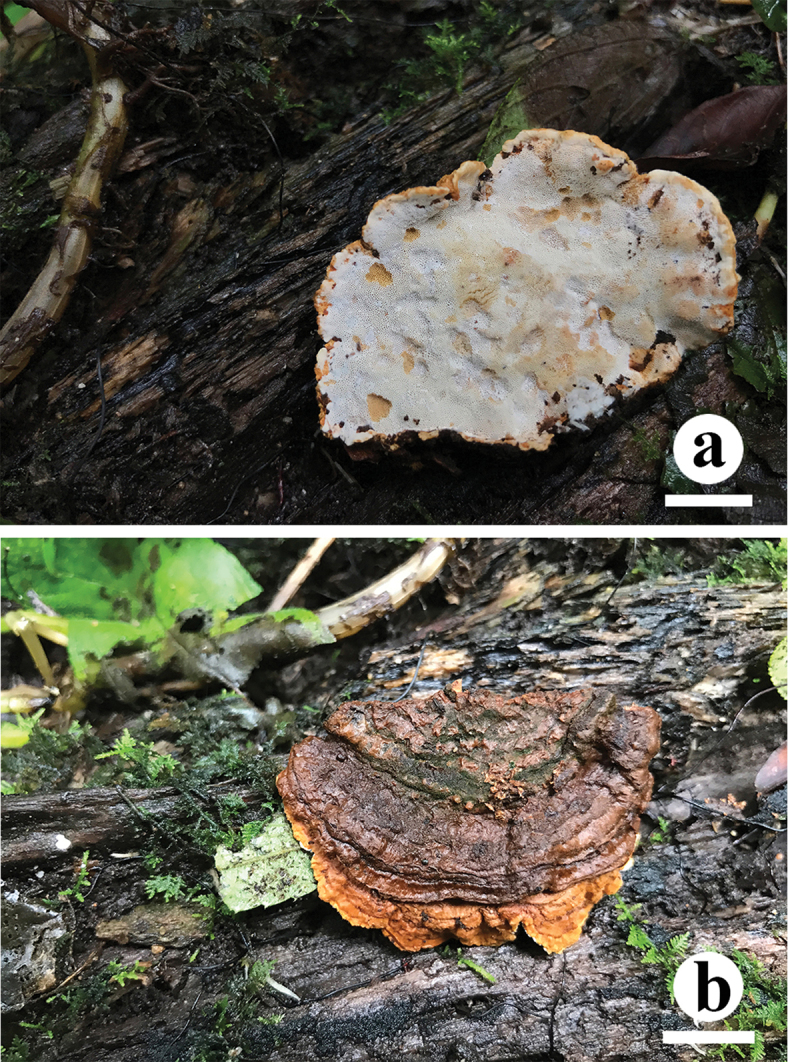
Basidiocarps of *Crassisporus microsporus*. Scale bars: 1 cm.

**Figure 10.**
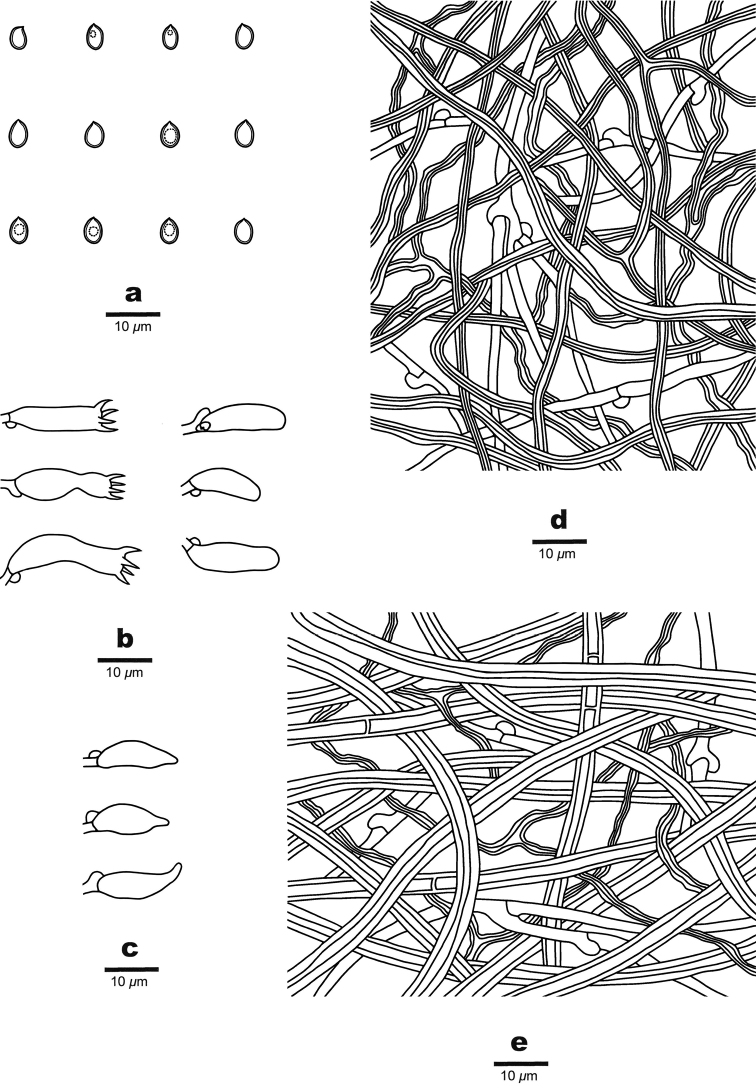
Microscopic structures of *Crassisporus microsporus* (drawn from the holotype) **A** basidiospores **B** basidia and basidioles **C** cystidioles **D** hyphae from trama **E** hyphae from context.

## Discussion

In the present study, *Crassisporus* is proposed based on morphological characters and phylogenetic analyses. In the ITS+nLSU analysis, *Crassisporus* was nested in the core polyporoid clade with strong support (100% MP, 100% ML, 1.00 BPP; Fig. 1). A further study based on combined ITS+nLSU+mtSSU+EF1-α+RPB2 sequences data indicated that *Crassisporus* grouped with *Haploporus* with low support, but formed a monophyletic lineage with a strong support (100% MP, 100% ML, 1.00 BPP; Fig. 2). Morphologically, *Crassisporus* is characterized by the combination of an annual growth habit, effused-reflexed to pileate basidiocarps, pale yellowish brown to yellowish brown, concentrically zonate or sulcate pilei, velutinate pileal surface, a trimitic hyphal system with clamped generative hyphae, tissues turning to dark in KOH, oblong to broadly ellipsoid, hyaline, smooth and slightly thick-walled basidiospores.

Morphologically, the four *Crassisporus* species can be easily distinguished from each other. *Crassisporus microsporus* differs from other species by its small pores (5–7 per mm), and small broadly ellipsoid basidiospores (4–5 × 3–3.7 μm). Except for *C. imbricatus*, *C. leucoporus*, *C. macroporus*, and *C. microsporus*, all have fusoid cystidioles in the hymenium; moreover, *C. imbricatus* produces imbricate basidiocarps. Previously, the type specimen of *C. imbricatus* was identified as *Coriolopsis byrsina* (Mont.) Ryvarden based on morphological characters (Li and Cui 2010). After careful examination of the basidiospores along with DNA sequences analyses, the specimen was found to represent an unknown taxon. Here, we describe it as a new species of *Crassisporus* based on morphological characters and phylogenetic analysis. *Crassisporus macroporus* may be confused with *C. leucoporus* due to their similarity in effused-reflexed to pileate basidiocarps and oblong ellipsoid basidiospores, but *C. leucoporus* is distinguished from *C. macroporus* by its smaller pores (3–4 per mm), white pore surface when fresh, and acyanophilous skeletal and binding hyphae.

Phylogenetically, *Haploporus* groups together with *Crassisporus* (Figs 1, 2), but the former differs by its annual to perennial growth habit, dimitic to trimitic hyphal system, and ornamented, cyanophilous basidiospores (Shen et al. 2016; Cui et al. 2019).

*Crassisporus* is similar to *Hexagonia* Fr. and *Neofomitella* Y.C. Dai, Hai J. Li & Vlasák, because these genera share pileate brown basidiocarps, a trimitic hyphal system with clamped generative hyphae, and tissues becoming dark in KOH. However, *Hexagonia* is distinguished from *Crassisporus* by its larger hexagonal pores and thin-walled basidiospores (Núñez and Ryvarden 2001). *Neofomitella* differs from *Crassisporus* in having crusted basidiocarps with the cuticle developing from base to margin, and smaller, thin-walled basidiospores (Li et al. 2014b).

Both *Perenniporia* Murrill and *Crassisporus* have hyaline and thick-walled basidiospores, but species of *Perenniporia* have cyanophilous, and variable dextrinoid skeletal hyphae. In addition, *Perenniporia* usually has truncate basidiospores (Gilbertson and Ryvarden 1987; Núñez and Ryvarden 2001; Zhao et al. 2013; Cui et al. 2019).

*Truncospora* Pilát is similar to *Crassisporus* in having pileate basidiocarps and variable presence of cystidioles. However, *Truncospora* is distinguished from *Crassisporus* by variable dextrinoid and cyanophilous skeletal hyphae and truncate, strongly dextrinoid basidiospores (Zhao and Cui 2013; Cui et al. 2019).

*Abundisporus* Ryvarden and *Crassisporus* share effused-reflexed or pileate basidiocarps, but *Abundisporus* differs by its pale-umber to deep-purplish-brown or greyish- to umber-brown context, dimitic hyphal system, and pale-yellowish basidiospores (Ryvarden 1998; Zhao et al. 2015; Cui et al. 2019).

*Perenniporiella* Decock & Ryvarden also has annual, pileate basidiocarps, and hyaline, thick-walled basidiospores, but it differs from *Crassisporus* in having a dimitic hyphal system (Decock and Ryvarden 2003).

*Grammothelopsis* Jülich is similar to *Crassisporus* in having thick-walled basidiospores; however, it differs from *Crassisporus* in its resupinate to effused basidiocarps with shallow irregular pores, and variable dextrinoid skeletal hyphae (Robledo and Ryvarden 2007; Zhao and Cui 2012).

### Key to species of *Crassisporus*

**Table:** 

1	Cystidioles absent	***C. imbricatus***
–	Cystidioles present	**2**
2	Basidiospores broadly ellipsoid	***C. microsporus***
–	Basidiospores oblong ellipsoid	**3**
3	Pore surface cream, buff to cinnamon-buff when fresh, pores 2–3 per mm	***C. macroporus***
–	Pore surface white when fresh, pores 3–4 per mm	***C. leucoporus***

## Supplementary Material

XML Treatment for Crassisporus

XML Treatment for Crassisporus imbricatus

XML Treatment for Crassisporus leucoporus

XML Treatment for Crassisporus macroporus

XML Treatment for Crassisporus microsporus
